# Impact of Patient Access to Internet Health Records on Glaucoma Medication: Randomized Controlled Trial

**DOI:** 10.2196/jmir.2795

**Published:** 2014-01-15

**Authors:** Kenji Kashiwagi, Shigeo Tsukahara

**Affiliations:** ^1^Faculty of MedicineUniversity of YamanashiChuoJapan

**Keywords:** Internet, glaucoma, intraocular pressure, personal health record, medication

## Abstract

**Background:**

Glaucoma is one of the leading causes of blindness. Reduction of intraocular pressure is the only proven way to prevent progression of glaucomatous optic neuropathy. The majority of glaucoma patients need to use antiglaucoma ophthalmic solutions over the course of their life. Thus, good adherence and persistency of glaucoma treatment are important factors for better glaucoma care.

**Objective:**

The purpose of this study was to investigate the impact of an Internet-based glaucoma care support system on glaucoma medication use.

**Methods:**

Patients were randomly divided into two groups. The non–Internet access (NIA) group consisted of patients who had access to the Internet-based glaucoma care support system during the 4-year period only when they were examined by ophthalmologists. The Internet access (IA) group consisted of patients who had the same Internet-based glaucoma care support system access as the NIA group for the first 2 years following enrollment but who were also given free access to the glaucoma care support system for the remaining 2 years. Changes in glaucoma medication use were investigated.

**Results:**

In total, 81 patients in the IA group and 90 patients in the NIA group satisfied the study protocol. The number of antiglaucoma ophthalmic solutions used during the study period significantly increased in the NIA group (*P*<.03) but not in the IA group. The percentages of patients with unchanged, increased, and decreased antiglaucoma ophthalmic solution use during the study period were 61.1% (55/90), 17.8% (16/90), and 3.3% (3/90), respectively, in the NIA group, and 56.8% (46/81), 8.6% (7/81), and 13.6% (11/81), respectively, in the IA group (*P*<.001). Internet access significantly shifted from an increasing intraocular pressure trend to a decreasing trend in the IA group (*P*=.002) among the patients who did not have any medication changes.

**Conclusions:**

Allowing patients to browse their medical data may reduce the use and improve the effectiveness of glaucoma medication.

**Trial Registration:**

UMIN-CTR Clinical Trial Number: UMIN000006982; https://upload.umin.ac.jp/cgi-open-bin/ctr/ctr.cgi?function=brows&action=brows&type=summary&recptno=R000008238&language=E (Archived by WebCite at http://www.webcitation.org/6MRPQeEAv).

## Introduction

Glaucoma is one of the leading causes of acquired blindness and reduction of intraocular pressure (IOP) is the only proven therapy. Glaucoma treatment generally consists of antiglaucoma ophthalmic solutions—medications that are required for long periods of time [1]. Recent studies have revealed that a number of patients fail to comply with proper glaucoma medication regimens [2] because many glaucoma patients lack any glaucoma-related symptoms.

Treatment adherence is a major concern in many chronic diseases [3], and improving patients’ understanding of their diseases has been found to be vital for appropriate treatment [4-6]. A variety of interventions have been used to increase understanding, including patient education during medical examinations, public lectures, distributing medical information pamphlets, and mass media advertising [7-11]. Okeke et al have reported that a multifaceted intervention significantly increased adherence to glaucoma medication during a 3-month trial [11]. Significant efforts have been devoted to promoting glaucoma education, which is considered critical to improving treatment effectiveness [8,12]. However, these efforts are expensive and require human resources. In addition, the effects of these efforts are transient [9,13,14], and more efficient and effective long-term patient education systems are needed.

In recent years, a personalized health record (PHR) system has been proposed. This system uses information and communication technology to allow patients to manage their own health conditions [15,16]. The main purpose of many proposed PHR systems is to share medical records among clinicians [16,17].

Unfortunately, the effects of PHR systems on clinical outcomes in previous studies have not been consistent. Miller et al reported that PHR-enabled self-management did not improve care in multiple sclerosis patients [18]. In their review, Tenforde et al concluded that the evidence supporting the clinical value of PHR remains limited, despite its potential to improve chronic disease management and patient outcomes [19].

Some previous studies have focused on the effects of PHR in glaucoma therapy [6,20]. Gray et al reported that individualized patient care improved glaucoma knowledge, pre-existing beliefs, and management of a daily eye drop regimen [20]. However, there is insufficient evidence on the benefits of medical record self-management in glaucoma treatment.

In 2005, we introduced an Internet-based glaucoma care support system (GSS) in Japan. This system allowed glaucoma patients to view their own medical records via the Internet at any time and from any location. The goal of this system was to deepen patient understanding of glaucoma and encourage active involvement in treatment.

In this study, we targeted patients who had used the GSS for more than 4 years and examined how access to the contents of their own glaucoma medical records (“Internet access”) affected their glaucoma treatment.

## Methods

### Ethics Statement

**Figure 1:**
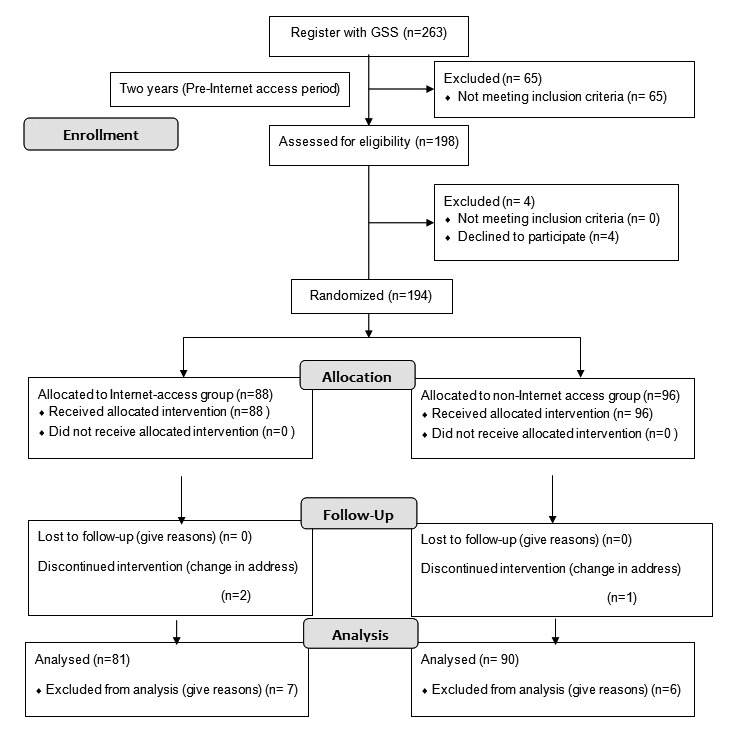
Flow diagram of study (GSS: glaucoma care support system).

This randomized, observer-blinded, prospective trial study was performed in accordance with the Helsinki Treaty and was approved by the University of Yamanashi Ethical Review Board. Written informed consent was obtained from all of the patients (see Figure 1 for the design of this study and Multimedia Appendix 1 for the CONSORT checklist; trial registration number UMIN000006982).

### Glaucoma Care Supporting System

**Figure 2:**
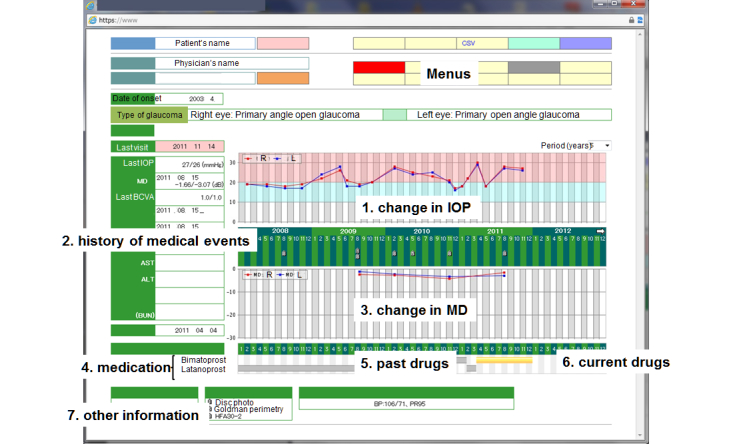
First page of GSS. History of medical events are marked on the calendar with numbers explained in a box (arrow). Changes in MD values indicate possibility of deterioration of glaucoma stage. Prescription details are shown by gray or colored bar, respectively. IOP: intraocular pressure, MD: mean deviation, R: right eye, L: left eye.

**Figure 3:**
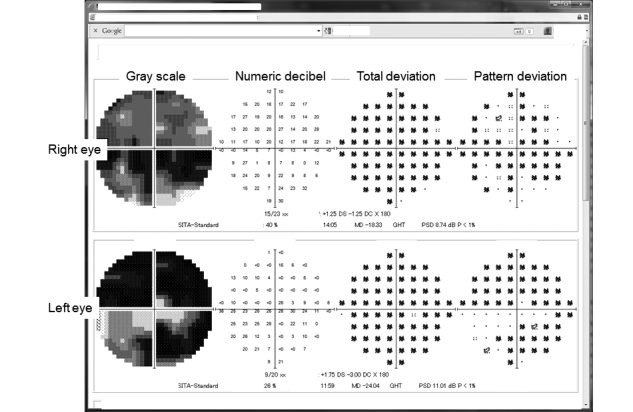
Results of the static visual field test using the Humphrey visual field test, including gray scale maps, numeric decibels, and the total and pattern deviation. MD: mean deviation, PSD: pattern standard deviation.

**Figure 4:**
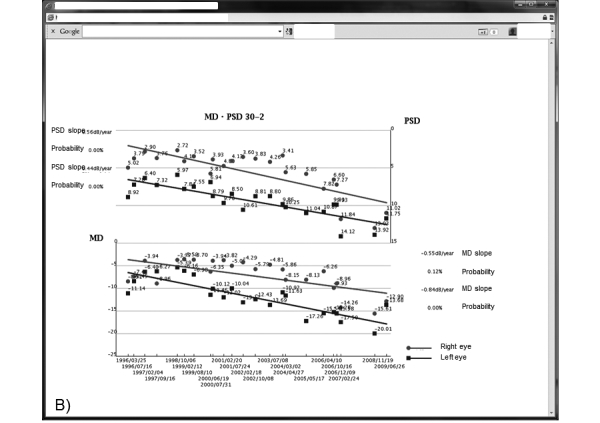
The PSD (upper graph) and MD slopes (lower graph) show the possibility of glaucoma progression with a probability analysis. MD: mean deviation, PSD: pattern standard deviation.

The GSS is a secure online PHR system. Registered patients can use the GSS to view their IOP values, visual field test results, current and prior medications, and other findings from their examination histories related to glaucoma. Figure 2 shows the first page of the GSS. Graphs are used to facilitate patient understanding of their medical records. Patients can view the detailed results of visual field tests on different pages (see Figures 3 and 4).

The GSS is based on the concept of an information security management system. The data are stored in a database server that is located in a facility at the University of Yamanashi. The registered GSS data are periodically updated by automated medical chart extraction or manual data entry by physicians and medical staff. All the data transferred from medical charts to the GSS are managed offline using locally developed data management programs. Patient registration for the GSS began in 2005, and approximately 1600 glaucoma patients were registered as of November 2011. Our plan was to allow registered patients to access their medical records by themselves. The GSS was the first PHR system routinely used in Japanese clinical ophthalmology care.

### Patients

Glaucoma patients who had been periodically treated at the University of Yamanashi Hospital Glaucoma Outpatient Clinic and expressed interest in registering with the GSS were subsequently registered for the GSS. Patients were informed of the benefits and risks of the GSS by the ophthalmologist and the medical staff. The patients were also informed that the registered medical records would not be available until security was well established. Registration for the GSS began in 2005. By 2008, only ophthalmologists were allowed to browse the registered medical records, and patients could view their registered medical records in the presence of the attending ophthalmologist at the clinic.

### Study Design

Internet access by patients began in 2008. We randomly selected patients for Internet access between January 2008 and December 2008. The inclusion criteria included the following: patients who had been registered in the GSS database for 2 years and who were diagnosed with either primary open angle glaucoma (POAG), normal tension glaucoma (NTG), or ocular hypertension (OH). The exclusion criteria included the following: patients who were under 20 years old at the time of registration; patients who had a history of intraocular surgery in both eyes; patients for whom accurate IOP measurement was deemed difficult; patients who had received oral glaucoma therapy, such as carbonic anhydrase inhibitors; patients who had a disease other than glaucoma that caused visual field defects; patients with a visual acuity of less than 20/60 or a mean deviation (MD) value lower than –20 dB in the worse eye, as determined by the Humphrey Field Analyzer (HFA) central 30-2 program (Zeiss Inc); and patients diagnosed with dementia whose use of GSS was judged by an ophthalmologist to be difficult.

After re-confirming the patients’ preferences for browsing their registered medical records, identification numbers and temporary passwords were sent to the patients to allow them Internet access. The patients began to browse the GSS after attending lectures by the system staff.

The users were asked to replace their temporary passwords and register their user names with the system. To maintain patient anonymity, no information that could identify a patient—such as name, age, gender, telephone number, home address, or business address—was accessible through the Internet.

The outcomes of the patients who were assigned to the Internet access (IA) group were compared with those of patients who viewed their medical records only with an ophthalmologist during their medical examinations (the non–Internet access group, or NIA group).

Patients who met any of the following criteria were excluded during the 2-year period post Internet access: those who died; had glaucoma surgery, including laser treatment; received oral glaucoma therapy, such as carbonic anhydrase inhibitors; developed a disease other than glaucoma that caused visual field disturbances or IOP changes; and developed severe visual impairment or dementia that caused GSS use to become difficult as judged by an ophthalmologist.

### Ophthalmological Examination

All of the patients visited the Glaucoma Outpatient Clinic at the University of Yamanashi Hospital approximately every 3 months. A best-corrected visual acuity (BCVA) measurement, an IOP measurement using a Goldmann applanation tonometer, a slit-lamp examination, and a fundus examination were performed as part of the routine examinations. The HFA visual field test was usually performed every 6-10 months and disc photography was performed every year. No differences were found in the ophthalmological examination protocol or follow-up schedule between the NIA and IA groups. The patient and the attending ophthalmologist discussed the results of the glaucoma examination using data displayed by the GSS. The ophthalmologists did not know whether the patient browsed the GSS data at home or elsewhere.

### Role of Changing Glaucoma Medication

Any glaucoma medication changes were determined by glaucoma specialists with the patient’s consent. The medical glaucoma treatment was determined by glaucoma specialists using a targeted IOP strategy. The specialists increased the number of glaucoma medication treatments in response to the following test results: two or more consecutive IOP values exceeding the target IOP, glaucomatous neuropathy deterioration suggested by a visual field test, and imaging tests focused on the optic nerve head and nerve fiber layer thickness. If two or more consecutive IOP values were sufficiently below the target IOP and the glaucoma specialists judged that reducing the glaucoma medication would not elevate the IOP over the target value, the glaucoma medication was reduced.

### Investigated Parameters

This study compared the amount of antiglaucoma ophthalmic solution used, MD values, and BCVA values between the two groups. In addition, we performed a subanalysis of IOP changes over the study period in those patients who did not report any changes in the amount of antiglaucoma ophthalmic solution used during the study. The IOP profiles were compared in the NIA and IA groups. IOP changes from the pre-IA to the post-IA period were also compared in the IA groups. The right eye was chosen for the analysis. If the right eye met exclusion criteria, then the left eye was subject to the analysis.

### Definition of Change in the Number of Antiglaucoma Ophthalmic Solutions Used

We analyzed changes in the amount of antiglaucoma ophthalmic solution used for individual patients and individual eyes. If the change in the amount of antiglaucoma ophthalmic solution used during the study period differed between the right and left eyes, the eye that showed greater change was used in the analysis. Any change in the concentration of the antiglaucoma ophthalmic solution used was considered to be a change in the antiglaucoma medication. However, a change to a different antiglaucoma ophthalmic solution having the same pharmacological action and similar IOP-reducing potential was not considered to be a change. For example, changing from a latanoprost ophthalmic solution to another prostaglandin-related antiophthalmic solution (other than an isopropyl unoprostone solution) and changing from a timolol maleate ophthalmic solution to another beta-blocker ophthalmic solution were not considered to be changes in antiglaucoma medication.

### Change in Medication Possession Ratio

We investigated the change in medication possession ratio (MPR) among patients whose medication was not changed through the study period. A useful definition of MPR as a parameter to measure adherence is the ratio of the days of prescription supply dispensed over the number of days between the first and last prescription refill [2,21,22]. The number of days of supplied medication was calculated from the actual number of drops per bottle, based on Fiscella’s report [23] or from the manufacturer’s estimates for the products, summarized by Friedman et al [2]. See Multimedia Appendix 2 for information on the days of supplied medication for the antiglaucoma ophthalmic solutions used.

### Statistical Analysis

The data were analyzed using the JMP 8.0 software package (SAS Institute Inc), and the values are presented as the mean and standard deviation (SD). Changes in the amount of antiglaucoma ophthalmic solutions used were analyzed using the Wilcoxon signed rank test or a contingency table analysis. The IOP, BCVA (expressed as logMAR), MD values of the HFA central 30-2 program, and MPR were compared between the IA and NIA groups using the Mann-Whitney U test. The IOP changes within a group were analyzed using the Student *t* test, and the type of glaucoma and patient gender were compared using Fisher’s exact probability test. The effect of Internet access on the IOP changing trend was analyzed using the analysis of covariance (ANCOVA) and Pearson correlation coefficient; *P*<.05 was considered to be statistically significant.

## Results

### Characteristics of Enrolled Patients

In total, 194 patients were randomly assigned to the two groups, and 81 IA and 90 NIA patients completed the study. Tables 1 and 2 show detailed information on patient dropouts and the characteristics of the patients who completed the study. The mean age, type of glaucoma, and gender did not differ significantly between the two groups. Both the IA and NIA patients had no significant changes in their BCVA and MD values obtained with the HFA central 30-2 program between the initial and final examinations.

**Table 1:** Characteristics of all enrolled patients.

Group	Start of IA			Details of dropout during the IA period			
	Patients, n	Age, years mean (SD)	Male, %	PE^a^	TLE^b^	Oral CAI^c^	Change in address
IA (Internet access)	88	62.6 (15.1)	62.6	2	2	1	2
NIA (non−Internet access)	96	64.6 (12.5)	87.1	1	2	2	1

^a^PE: phacoemulcification.

^b^TLE: trabeculectomy.

^c^CAI: carbonic anhydrase inhibitor.

**Table 2:** Characteristics of completed patients (numbers in parentheses are standard deviation).

Group	Patients, n	Age, years mean (SD)	Type of glaucoma^a^ (POAG:NTG:OH)	Male (%)	Initial BCVA^b^	Final BCVA	Initial MD^c^ (dB)	Final MD (dB)
IA (Internet Access)	81	61.8 (15.3)	36:42:3	63.0	0.09 (0.39)	0.1 (0.39)	–6.47 (7.36)	–7.16 (7.66)
NIA (Non−Internet Access)	90	63.4 (11.8)	38:47:5	54.4	0.03 (0.22)	0.03 (0.22)	–6.27 (7.36)	–6.83 (7.43)

^a^POAG: primary open angle glaucoma, NTG: normal tension glaucoma, OH: ocular hypertension.

^b^BCVA: best-corrected visual acuity.

^c^MD: mean deviation.

### Changes in the Amount of Anti-Glaucoma Ophthalmic Solution Used

The number of antiglaucoma ophthalmic solutions used at the initial examination in the IA group was mean 1.2 (SD 1.2) in the right eye and mean 1.3 (SD 1.1) in the left eye, while the same number in the NIA group was mean 1.0 (SD 1.0) in the right eye and mean 1.1 (SD 1.0) in the left eye. The number of antiglaucoma ophthalmic solutions used did not differ significantly between the IA and NIA groups. In the NIA group at the final examination, it was mean 1.2 (SD 1.1) in the right eye and mean 1.2 (SD 1.0) in the left eye, which were significantly greater than the values at the initial examination (*P*<.001 for the right eye, *P*=.03 for the left eye, Wilcoxon signed-rank test). In contrast, the number of antiglaucoma ophthalmic solutions used in the IA group decreased by mean 0.1 (SD 0.4) in the right eye and by mean 0.1 (SD 0.6) in the left eye. The changes in the number of antiglaucoma ophthalmic solutions used in the NIA and IA groups were mean 0.3 (SD 0.4) and mean –0.1 (SD 0.4), respectively, which is significantly different (*P*<.001, Wilcoxon signed-rank test). None of the patients reported using less antiglaucoma ophthalmic solution in one eye and more in the other eye during the investigation period.

### Distribution of Change in the Amount of Antiglaucoma Ophthalmic Solution Used

**Figure 5:**
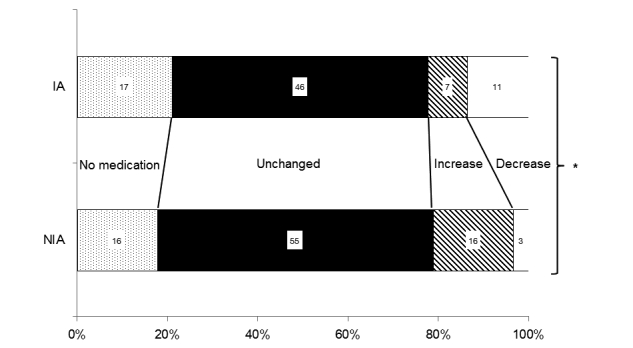
Changes in glaucoma medication during the study period (*P=.008, 2 x 4 contingency table analysis).

Figure 5 shows the distribution of the medication used by the glaucoma patients during the course of the study. In the NIA group (n=90), the amount of antiglaucoma ophthalmic solution used did not change in 55 patients (61.1%), increased in 16 patients (17.8%), and decreased in 3 patients (3.3%). No medications were administered to 16 patients (17.8%). In the IA group (n=81), the amount of antiglaucoma ophthalmic solution used did not change in 46 patients (56.8%), increased in 7 patients (8.6%), and decreased in 11 patients (13.6%). No medications were administered to 17 patients (21.0%). Compared to the NIA group, 10.3% more IA patients reported using decreased number of antiglaucoma ophthalmic solutions and 9.2% fewer patients reported using increased number of antiglaucoma ophthalmic solutions. A significant difference was found in the distribution of medications received between the two groups of glaucoma patients (*P*=.008, contingency table analysis).

### Changes in Intraocular Pressure Values

IOP values at the first examination of the IA and NIA groups were mean 15.3 (SD 4.2) mmHg and mean 15.1 (SD 3.1) mmHg, respectively (*P*=.69). IOP values at the last examination of the IA and NIA groups were mean 14.6 (SD 3.6) mmHg and mean 15.1 (SD 3.3) mmHg, respectively (*P*=.26). Changes in IOP over the study period were not significant in either the IA (*P*=.23) or the NIA group (*P*=.83).

### Analysis of the Patients Whose Antiglaucoma Ophthalmic Solution Use Did Not Change During the Study Period

We examined the patients whose antiglaucoma ophthalmic solution use did not change over the course of the study. The subjects included 46 patients in the IA group and 55 patients in the NIA group. The distribution of the cases is shown in Table 3. The two groups did not differ significantly by age, gender, type of glaucoma, or the amount of antiglaucoma ophthalmic solutions used. The two groups also did not differ significantly by their BCVA and MD values measured at the beginning of the study or by changes in their BCVA and MD values during the study period. See Multimedia Appendices 3 and 4 for information on the antiglaucoma ophthalmic solutions used.

**Table 3:** Characteristics of patients whose antiglaucoma ophthalmic solution use did not change during the study period (numbers in parentheses are standard deviation).

Group	Patients, n	Age, years mean (SD)	Type of glaucoma^a^ (POAG:NTG:OH)	Male, %	# of antiglaucoma ophthalmic solution	Initial BCVA^b^	Final BCVA	Initial MD^c^ (dB)	Final MD (dB)
IA (Internet Access)	46	63.7 (12.8)	21:24:1	66.7	1.5 (0.9)	0.08 (0.38)	0.10 (0.40)	–6.01 (7.32)	–7.04 (7.61)
NIA (Non−Internet Access)	55	66.4 (10.7)	21:32:2	61.1	1.2 (0.8)	0.09 (0.31)	0.10 (0.25)	–6.67 (7.24)	–8.07 (7.94)

^a^POAG: primary open angle glaucoma, NTG: normal tension glaucoma, OH: ocular hypertension.

^b^BCVA: best-corrected visual acuity.

^c^MD: mean deviation.

### Internet Access and Intraocular Pressure Changes

The IOP values in the IA and NIA groups were mean 15.2 (SD 4.2) mmHg and mean 14.5 (SD 2.1) mmHg at the first examination, respectively. No significant IOP differences were observed between the IA and NIA groups (*P*=.31).

Differences in the IOP values between the first examination and the final examination in the IA group and NIA group were mean 0.2 (SD 1.4) mmHg (*P*=.32) and –0.8 (SD 3.0) mmHg (*P*=.10), respectively. The IA group showed a tendency toward IOP reduction during the study period.

**Figure 6:**
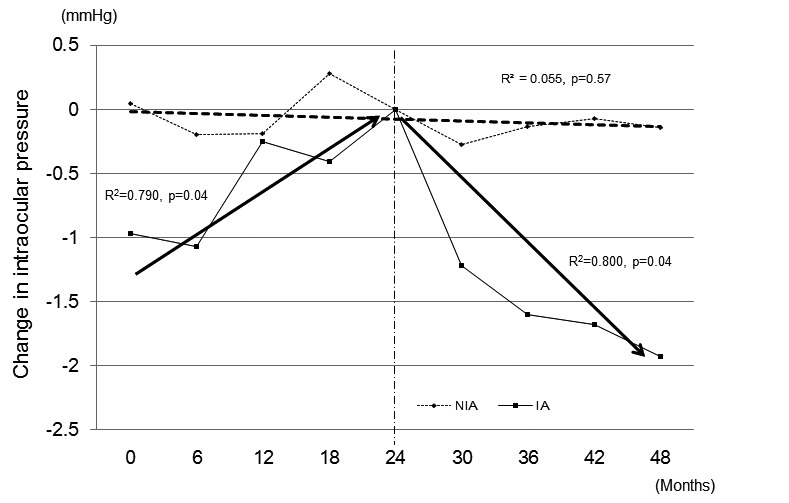
A change in the intraocular pressure at 24 months after the start of the study was defined as zero mmHg. IA: Internet access group, NIA: non-Internet access group.

In the IA group, the IOP of both eyes changed significantly between the pre-IA period and the post-IA period. Figure 6 shows the changing IOP profiles during the study period. In the IA group, the IOP was increasing before the start of Internet access (*R* ^2^=.790, *P*=.04) and decreased after the start of Internet access (*R* ^2^=.800, *P*=.04). By contrast, no significant IOP changes were observed during the study period in the NIA group (*R* ^2^=.055, *P*=.57). The ANCOVA showed that Internet access significantly shifted from an increasing IOP trend to a decreasing trend in the IA group (*P*=.002). The IOP increased by mean 0.1 (SD 0.1) mmHg/month during the pre-IA period, while the IOP decreased by mean 0.1 (SD 0.1) mmHg/month during the post-IA period.

### Change in Medication Possession Ratio

In the IA group, the MPRs before and after the start of Internet access were mean 82.3% (SD 30.7) and 91.1% (SD 40.3), respectively, which indicates a significant improvement (*P*=.03). In the NIA group, the MPRs before and after the start of Internet access were mean 84.0% (SD 28.5) and 82.9% (SD 31.1), respectively, which does not indicate significant improvement (*P*=.56).

## Discussion

### Principal Findings

The utility of patient education and medical information services has been previously discussed [7,8]. Despite multiple efforts, however, there is insufficient evidence supporting the utility of patient education and medical information services in glaucoma treatment [8,24,25]. The current study showed that allowing glaucoma patients to self-browse their clinical data contributed to two favorable effects: using less antiglaucoma ophthalmic solution and improving the effectiveness of the medication in reducing IOP.

It was common for the patients to increase their medication use during the treatment period. Indeed, 17.8% (16/90) of the NIA patients increased their glaucoma medications during the study period, while only 8.6% (7/81) of the IA patients did. In addition, 13.6% (11/81) of the IA patients reduced their medications during the study period. Notably, among the patients whose antiglaucoma ophthalmic solution use did not change during the study period, the Internet access group shifted from an increasing IOP trend to a decreasing trend. It is possible that the amount of antiglaucoma ophthalmic solutions used was increased in the patients who did not start Internet access. It is unclear whether the current outcomes were the direct results of Internet access or were due to other mechanisms, such as improved patient-physician communication during office visits. Improving adherence may contribute to these outcomes, although we were unable to monitor changes in adherence using subjective and quantitative methods, such as electronic monitoring systems, which are not available in Japan.

The current study showed that Internet access significantly improved the MPR among patients whose medication was not changed during the study and that Internet access may contribute to a reduction in IOP over time. The MPR in the current study is higher than that in Friedman’s report [2]. A possible explanation for this difference could be that the understanding of glaucoma and the motivation for glaucoma treatment may have been stronger for the patients in this study than those in the previous study. In the present study, patients had been treated for glaucoma before their enrollment and were willing to use the GSS.

**Figure 7:**
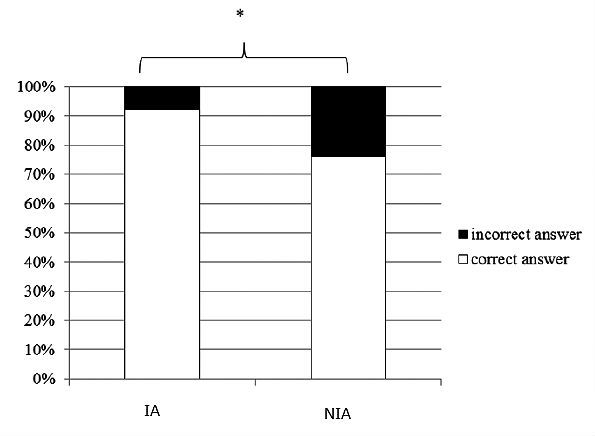
Comparison of the rates of glaucoma patients’ understanding of their target intraocular pressure (*P<.001, Fisher exact test).

**Figure 8:**
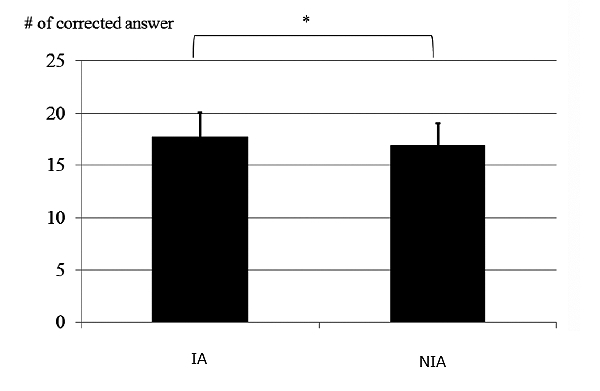
Comparison of number of correct answers to questions regarding glaucoma (*P=.007, Wilcoxon signed-rank test, RE).

Health literacy is an important consideration in using electronic PHRs appropriately. We examined glaucoma literacy by administering a questionnaire to another set of patients who had been registered with the GSS for more than 2 years [26]. The patients who had been self-checking their registered data for more than 1 year exhibited a much better understanding of aspects of their glaucoma, such as their glaucoma severity, IOP values, and medications, than did the patients who had been provided their registered data by the ophthalmologists (see Figures 7 and 8). The IA patients in the current study may have improved their eye health literacy by checking their data through the Internet.

Although the current study demonstrated the effects of Internet access on glaucoma treatment, these effects are not consistent with those observed in previous studies. In their review, Tenforde et al [19] concluded that despite PHR’s potential to improve chronic disease management and patient outcomes, the evidence supporting the clinical value of PHR remains limited. Many of the previous studies have enrolled patients with diabetes mellitus or other systemic diseases in which interventions may influence clinical outcomes through complicated processes. By contrast, glaucoma is a disease in which proper medication is the only intervention proven to influence IOP control. This difference could be a possible explanation for inconsistencies with previous studies.

We used a prospective, randomized study design, but the NIA patients were slightly older than the IA patients. The effect of age on adherence is controversial. Dietlein et al have reported that adherence to therapy with antiglaucoma ophthalmic solutions deteriorated with age [27,28], while other previous studies have reported that younger patients have poor adherence [29,30]. In the present study, no significant differences were found in the MD value, the number of antiglaucoma ophthalmic solutions used, or the BCVA value, suggesting that there were no marked differences in glaucoma severity between the two groups. Although we eliminated the patients who met the exclusion criteria, no significant differences were found in the reasons for loss to follow up between the two groups. Altogether, the influence of any factors, except for Internet access, between the two groups may have been limited.

### Limitations

The patients in this study were GSS users. Given that many patients cannot use a personal computer or the Internet due to their age, poverty, or other reasons, it is possible that the patients in this study are not representative of general glaucoma patients. The present study did not examine how frequently the patients accessed the GSS. Therefore, further investigations should be performed to verify the relationship between the frequency of use and the improvements in glaucoma treatment associated with using the system.

### Conclusion

The current study confirmed that patients’ understanding of their glaucoma care status may play an important role in better management.

## Multimedia Appendix 1

CONSORT-EHEALTH Checklist V1.6.2 [31].

## Multimedia Appendix 2

Days of supplied medication for antiglaucoma ophthalmic solutions used.

## Multimedia Appendix 3

Antiglaucoma ophthalmic solutions used (1).

## Multimedia Appendix 4

Antiglaucoma ophthalmic solutions used (2).

## Abbreviations

ANCOVA: analysis of covariance
BCVA: best-corrected visual acuity
CAI: carbonic anhydrase inhibitor
GSS: glaucoma care support system
HFA: Humphrey Field Analyzer
IA: Internet access
IOP: intraocular pressure
MD: mean deviation
MPR: medication possession ratio
NIA: non–Internet access
NTG: normal tension glaucoma
OH: ocular hypertension
PE: phacoemulcification
PHR: personalized health record
POAG: primary open angle glaucoma
TLE: trabeculectomy

## References

[1] Quigley HA (2011). Glaucoma. Lancet.

[2] Friedman DS, Quigley HA, Gelb L, Tan J, Margolis J, Shah SN, Kim EE, Zimmerman T, Hahn SR (2007). Using pharmacy claims data to study adherence to glaucoma medications: methodology and findings of the Glaucoma Adherence and Persistency Study (GAPS). Invest Ophthalmol Vis Sci.

[3] Vanelli M, Pedan A, Liu N, Hoar J, Messier D, Kiarsis K (2009). The role of patient inexperience in medication discontinuation: a retrospective analysis of medication nonpersistence in seven chronic illnesses. Clin Ther.

[4] Friedman DS, Hahn SR, Gelb L, Tan J, Shah SN, Kim EE, Zimmerman TJ, Quigley HA (2008). Doctor-patient communication, health-related beliefs, and adherence in glaucoma results from the Glaucoma Adherence and Persistency Study. Ophthalmology.

[5] Juzych MS, Randhawa S, Shukairy A, Kaushal P, Gupta A, Shalauta N (2008). Functional health literacy in patients with glaucoma in urban settings. Arch Ophthalmol.

[6] Muir KW, Ventura A, Stinnett SS, Enfiedjian A, Allingham RR, Lee PP (2012). The influence of health literacy level on an educational intervention to improve glaucoma medication adherence. Patient Educ Couns.

[7] Gray TA, Fenerty C, Harper R, Lee A, Spencer AF, Campbell M, Henson DB, Waterman H (2010). Preliminary survey of educational support for patients prescribed ocular hypotensive therapy. Eye (Lond).

[8] Hahn SR, Friedman DS, Quigley HA, Kotak S, Kim E, Onofrey M, Eagan C, Mardekian J (2010). Effect of patient-centered communication training on discussion and detection of nonadherence in glaucoma. Ophthalmology.

[9] Kharod BV, Johnson PB, Nesti HA, Rhee DJ (2006). Effect of written instructions on accuracy of self-reporting medication regimen in glaucoma patients. J Glaucoma.

[10] Okeke CO, Quigley HA, Jampel HD, Ying GS, Plyler RJ, Jiang Y, Friedman DS (2009). Interventions improve poor adherence with once daily glaucoma medications in electronically monitored patients. Ophthalmology.

[11] Rees G, Leong O, Crowston JG, Lamoureux EL (2010). Intentional and unintentional nonadherence to ocular hypotensive treatment in patients with glaucoma. Ophthalmology.

[12] Lunnela J, Kääriäinen M, Kyngäs H (2010). The views of compliant glaucoma patients on counselling and social support. Scand J Caring Sci.

[13] Goudswaard AN, Stolk RP, Zuithoff NPA, de Valk HW, Rutten GE (2004). Long-term effects of self-management education for patients with Type 2 diabetes taking maximal oral hypoglycaemic therapy: a randomized trial in primary care. Diabet Med.

[14] Pladevall M, Brotons C, Gabriel R, Arnau A, Suarez C, de la Figuera M, Marquez E, Coca A, Sobrino J, Divine G, Heisler M, Williams LK, Writing Committee on behalf of the COM99 Study Group (2010). Multicenter cluster-randomized trial of a multifactorial intervention to improve antihypertensive medication adherence and blood pressure control among patients at high cardiovascular risk (the COM99 study). Circulation.

[15] Fernandez-Luque L, Karlsen R, Bonander J (2011). Review of extracting information from the Social Web for health personalization. J Med Internet Res.

[16] Mandl KD, Kohane IS (2008). Tectonic shifts in the health information economy. N Engl J Med.

[17] Schargus M, Grehn F, Glaucocard Workgroup (2008). The European Glaucoma Society Glaucocard project: improved digital documentation of medical data for glaucoma patients based on standardized structured international datasets. Graefes Arch Clin Exp Ophthalmol.

[18] Miller DM, Moore SM, Fox RJ, Atreja A, Fu AZ, Lee JC, Saupe W, Stadtler M, Chakraborty S, Harris CM, Rudick RA (2011). Web-based self-management for patients with multiple sclerosis: a practical, randomized trial. Telemed J E Health.

[19] Tenforde M, Jain A, Hickner J (2011). The value of personal health records for chronic disease management: what do we know?. Fam Med.

[20] Gray TA, Fenerty C, Harper R, Spencer AF, Campbell M, Henson DB, Waterman H (2012). Individualised patient care as an adjunct to standard care for promoting adherence to ocular hypotensive therapy: an exploratory randomised controlled trial. Eye (Lond).

[21] Sikka R, Xia F, Aubert RE (2005). Estimating medication persistency using administrative claims data. Am J Manag Care.

[22] Andrade SE, Kahler KH, Frech F, Chan KA (2006). Methods for evaluation of medication adherence and persistence using automated databases. Pharmacoepidemiol Drug Saf.

[23] Fiscella RG, Green A, Patuszynski DH, Wilensky J (2003). Medical therapy cost considerations for glaucoma. Am J Ophthalmol.

[24] Amro R, Cox CL, Waddington K, Siriwardena D (2010). Glaucoma expert patient programme and ocular hypotensive treatment. Br J Nurs.

[25] Watkinson S (2010). Improving care of chronic open angle glaucoma. Nurs Older People.

[26] Kashiwagi K (2011). Effects of Web-based medical data providing on disease literacy of glaucoma patients. Effects of Web-based medical data providing on disease literacy of glaucoma patients J Jpn Telemed Telecare.

[27] Dietlein TS, Jordan JF, Lüke C, Schild A, Dinslage S, Krieglstein GK (2008). Self-application of single-use eyedrop containers in an elderly population: comparisons with standard eyedrop bottle and with younger patients. Acta Ophthalmol.

[28] Lacey J, Cate H, Broadway DC (2009). Barriers to adherence with glaucoma medications: a qualitative research study. Eye (Lond).

[29] Ashaye AO, Adeoye AO (2008). Characteristics of patients who drop out from a glaucoma clinic. J Glaucoma.

[30] Ngan R, Lam DL, Mudumbai RC, Chen PP (2007). Risk factors for noncompliance with follow-up among normal-tension glaucoma suspects. Am J Ophthalmol.

[31] Eysenbach G, CONSORT-EHEALTH Group (2011). CONSORT-EHEALTH: improving and standardizing evaluation reports of Web-based and mobile health interventions. J Med Internet Res.

