# Supplementary material for: Impact of Patient Access to Internet Health Records on Glaucoma Medication: Randomized Controlled Trial
Source: J Med Internet Res. 2014 Jan 15;16(1):e15. doi: 10.2196/jmir.2795 (PMC3906702; doi:10.2196/jmir.2795)
Supplement: Supplementary file 1 [file jmir_v16i1e15_app1.pdf]

CONSORT-EHEALTH Checklist V1.6.2 Report

(based on CONSORT-EHEALTH V1.6), available at [http://tinyurl.com/consort-ehealth-v1-6].

2795

Date completed

11/6/2013 11:51:44

by

Kenji Kashiwagi

Impact of Patient Access to Medical Records via the Internet on Glaucoma Medication: Randomized Controlled Trial

TITLE

1a-i) Identify the mode of delivery in the title

via the Internet

1a-ii) Non-web-based components or important co-interventions in title

1a-iii) Primary condition or target group in the title

Glaucoma

ABSTRACT

1b-i) Key features/functionalities/components of the intervention and comparator in the METHODS section of the ABSTRACT

To compare the impact of an Internet-based glaucoma care support system (GSS) on glaucoma medication use between randomly divided into two groups: the non-Internet-access (NIA) group, which consisted of patients who had access to the GSS during the four-year period only when they were examined by ophthalmologists; and the Internet access (IA) group, which consisted of patients who had the same GSS access as the NIA group for the first two years following enrollment but who were also given free access to the GSS for the remaining two years.

1b-ii) Level of human involvement in the METHODS section of the ABSTRACT

Eighty-one patients in the IA group and 90 patients in the NIA group met the study criteria.

1b-iii) Open vs. closed, web-based (self-assessment) vs. face-to-face assessments in the METHODS section of the ABSTRACT

The non-Internet-access (NIA) group, which consisted of patients who had access to the GSS during the four-year period only when they were examined by ophthalmologists; and the Internet access (IA) group, which consisted of patients who had the same GSS access as the NIA group for the first two years following enrollment but who were also given free access to the GSS for the remaining two years.

1b-iv) RESULTS section in abstract must contain use data

Eighty-one patients in the IA group and 90 patients in the NIA group met the study criteria. The number of anti-glaucoma ophthalmic solution used during the study period significantly increased in the NIA group (($p<0.001$ for the right eye, $p= 0.03$ for the left eye) but not in the IA group. The percentages of patients with unchanged, increased, and decreased anti-glaucoma ophthalmic solution use during the study period were, 61.1%, 17.8%, and 3.3%, respectively, in the NIA group, and 56.8%, 8.6%, and 13.6%, respectively, in the IA group ($p < 0.001$). The Internet access significantly altered the IOP trend from increasing to decreasing in the IA group ($P=0.002$) among the patients who did not have any medication changes.

1b-v) CONCLUSIONS/DISCUSSION in abstract for negative trials

Allowing patients to browse their medical data may reduce the use and improve the effectiveness of glaucoma medication.

INTRODUCTION

2a-i) Problem and the type of system/solution

Glaucoma is one of the leading causes of acquired blindness and a reduction of the intra-ocular pressure (IOP) is the only proven therapy.
A recent study has revealed that a number of patients fail to comply with proper glaucoma medication regimens.
We introduced the Internet-based glaucoma care support system (GSS). This system allows glaucoma patients to view their own medical records via the Internet at any time and from any location. We hypothesized that patients self-browsing their clinical data through the GSS may improve the effectiveness of medication in reducing their IOP.

2a-ii) Scientific background, rationale: What is known about the (type of) system
Glaucoma treatment generally consists of anti-glaucoma ophthalmic solutions, and this medication is required for long periods of time. A recent study has revealed that a number of patients fail to comply with proper glaucoma medication regimens.

METHODS

3a) CONSORT: Description of trial design (such as parallel, factorial) including allocation ratio
We hypothesized that patients self-browsing their clinical data through the GSS may improve the effectiveness of medication in reducing their IOP.

3b) CONSORT: Important changes to methods after trial commencement (such as eligibility criteria), with reasons
None

3b-i) Bug fixes, Downtimes, Content Changes

4a) CONSORT: Eligibility criteria for participants
he inclusion criteria included the following: patients who had been registered in the GSS database for two years; and patients who were diagnosed with either primary open angle glaucoma (POAG), normal tension glaucoma (NTG), or ocular hypertension (OH). The exclusion criteria included the following: patients who were under twenty years of age at the time of registration; patients who had a history of intraocular surgery in both eyes; patients for whom accurate IOP measurement was deemed difficult; patients who had received oral glaucoma therapy, such as carbonic anhydrase inhibitors; patients who had a disease other than glaucoma that caused visual field defects; patients with a visual acuity of less than 20/60 or a mean deviation (MD) value lower than -20 dB in the worst eye, as determined by the Humphrey Field Analyzer (HFA) central 30-2 program (Zeiss Inc., Dublin, CA, USA); and patients diagnosed with dementia whose use of the GSS was judged to be difficult by an ophthalmologist.

4a-i) Computer / Internet literacy

4a-ii) Open vs. closed, web-based vs. face-to-face assessments:
The patients were randomly divided into two groups: the non-Internet-access (NIA) group, which consisted of patients who had access to the GSS during the four-year period only when they were examined by ophthalmologists; and the Internet access (IA) group, which consisted of patients who had the same GSS access as the NIA group for the first two years following enrollment but who were also given free access to the GSS for the remaining two years.

4a-iii) Information giving during recruitment
Glaucoma patients who had been periodically treated at the University of Yamanashi Hospital Glaucoma Outpatient Clinic and who expressed an interest in registering with the GSS were subsequently registered for the GSS. Patients were informed of the benefits and risks of the GSS by the ophthalmologist and the medical staff. The patients were also informed that patients could access the registered medical records by themselves until security was well established. Registration for the GSS began in 2005. By 2008, only ophthalmologists were allowed to browse the registered medical records and the patients were required to view their registered medical records in the presence of the attending ophthalmologist at the clinic.

4b) CONSORT: Settings and locations where the data were collected
The data are stored in a database server that is located in a facility at the University of Yamanashi.

4b-i) Report if outcomes were (self-)assessed through online questionnaires
NA

4b-ii) Report how institutional affiliations are displayed
The data are stored in a database server that is located in a facility at the University of Yamanashi.

5) CONSORT: Describe the interventions for each group with sufficient details to allow replication, including how and when they were actually administered

5-i) Mention names, credential, affiliations of the developers, sponsors, and owners
The GSS is based on the concept of an information security management system. The data are stored in a database server that is located in a facility at the University of Yamanashi. The registered GSS data are periodically updated by an automated medical chart extraction or manual data entry by physicians and medical staff.

5-ii) Describe the history/development process

The data are stored in a database server that is located in a facility at the University of Yamanashi. The registered GSS data are periodically updated by an automated medical chart extraction or manual data entry by physicians and medical staff. All of the data transferred from medical charts to the GSS are managed offline using locally developed data management programs. Patient registration for the GSS began in 2005, and approximately 1,600 glaucoma patients had been registered by November 2011. Our plan was to allow registered patients to access their medical records by themselves. The GSS was the first patient-access enabled EHR system routinely used in Japanese clinical ophthalmology care.

5-iii) Revisions and updating

5-iv) Quality assurance methods

The registered GSS data are periodically updated by an automated medical chart extraction or manual data entry by physicians and medical staff. All of the data transferred from medical charts to the GSS are managed offline using locally developed data management programs.

5-v) Ensure replicability by publishing the source code, and/or providing screenshots/screen-capture video, and/or providing flowcharts of the algorithms used

5-vi) Digital preservation

5-vii) Access

Our plan was to allow registered patients to access their medical records by themselves. The GSS was the first patient-access enabled EHR system routinely used in Japanese clinical ophthalmology care.

5-viii) Mode of delivery, features/functionalities/components of the intervention and comparator, and the theoretical framework

In 2005, we introduced the Internet-based glaucoma care support system (GSS) in Japan; this system was developed and is owned by the University of Yamanashi.

The GSS was the first patient-access enabled EHR system routinely used in Japanese clinical ophthalmology care.

5-ix) Describe use parameters

A best-corrected visual acuity (BCVA) measurement, an IOP measurement using a Goldmann applanation tonometer, a slit-lamp examination, and a fundus examination were performed as a part of the routine examinations. The HFA visual field test was usually performed every six to ten months, and disc photography was performed every year.

5-x) Clarify the level of human involvement

The majority of these data were automatically entered from the electronic chart system, except for the descriptive data supplied by ophthalmologists, which maintains the quality of this system.

5-xi) Report any prompts/reminders used

NA

5-xii) Describe any co-interventions (incl. training/support)

NA

6a) CONSORT: Completely defined pre-specified primary and secondary outcome measures, including how and when they were assessed

We hypothesized that patients self-browsing their clinical data through the GSS may improve the effectiveness of medication in reducing their IOP.

6a-i) Online questionnaires: describe if they were validated for online use and apply CHERRIES items to describe how the questionnaires were designed/deployed

6a-ii) Describe whether and how “use” (including intensity of use/dosage) was defined/measured/monitored

6a-iii) Describe whether, how, and when qualitative feedback from participants was obtained

6b) CONSORT: Any changes to trial outcomes after the trial commenced, with reasons

None

7a) CONSORT: How sample size was determined
7a-i) Describe whether and how expected attrition was taken into account when calculating the sample size

7b) CONSORT: When applicable, explanation of any interim analyses and stopping guidelines
NA
8a) CONSORT: Method used to generate the random allocation sequence
In detail, the patients whose last identification number digit was even or odd were assigned to the Internet access (IA) group or the non-Internet access (NIA) group, respectively.
8b) CONSORT: Type of randomisation; details of any restriction (such as blocking and block size)
In detail, the patients whose last identification number digit was even or odd were assigned to the Internet access (IA) group or the non-Internet access (NIA) group, respectively.
9) CONSORT: Mechanism used to implement the random allocation sequence (such as sequentially numbered containers), describing any steps taken to conceal the sequence until interventions were assigned
The ophthalmologists were masked with respect to which study group their patients were in and prohibited from asking whether the patient browsed the GSS data at home or elsewhere.
10) CONSORT: Who generated the random allocation sequence, who enrolled participants, and who assigned participants to interventions
Glaucoma patients who had been periodically treated at the University of Yamanashi Hospital Glaucoma Outpatient Clinic and who expressed an interest in registering with the GSS were subsequently registered for the GSS.
11a) CONSORT: Blinding - If done, who was blinded after assignment to interventions (for example, participants, care providers, those assessing outcomes) and how
11a-i) Specify who was blinded, and who wasn't
The ophthalmologists were masked with respect to which study group their patients were in and prohibited from asking whether the patient browsed the GSS data at home or elsewhere.
11a-ii) Discuss e.g., whether participants knew which intervention was the "intervention of interest" and which one was the "comparator"

11b) CONSORT: If relevant, description of the similarity of interventions
NA
12a) CONSORT: Statistical methods used to compare groups for primary and secondary outcomes
The data were analyzed using the JMP 8.0 software package (SAS Institute Inc., Cary, NC), and the values are presented as the mean ± standard deviation. Changes in the amount of anti-glaucoma ophthalmic solutions used were analyzed using the Wilcoxon ranked-sign test or a 2 x 4 contingency table analysis. The IOP, BCVA (expressed as logMAR), MD values of the HFA central 30-2 program, and MPR were compared between the IA and NIA groups using the Mann-Whitney U-test. The IOP changes between pre-IA period and the post-IA period within a group were analyzed using Student's t-test. The type of glaucoma or patient gender were compared between the IA and NIA groups using 2 x 3 contingency table analysis or Fisher's exact probability test. Pearson's correlation coefficient was used to analyze the correlation between follow up period and IOP change. The effect of the Internet access on the IOP trend alterations was analyzed using the analysis of covariance (ANCOVA). $P<0.05$ was considered to be statistically significant.
12a-i) Imputation techniques to deal with attrition / missing values
The exclusion criteria included the following: patients who were under twenty years of age at the time of registration; patients who had a history of intraocular surgery in both eyes; patients for whom accurate IOP measurement was deemed difficult; patients who had received oral glaucoma therapy, such as carbonic anhydrase inhibitors; patients who had a disease other than glaucoma that caused visual field defects; patients with a visual acuity of less than 20/60 or a mean deviation (MD) value lower than -20 dB in the worst eye, as determined by the Humphrey Field Analyzer (HFA) central 30-2 program (Zeiss Inc., Dublin, CA, USA); and patients diagnosed with dementia whose use of the GSS was judged to be difficult by an ophthalmologist.
12b) CONSORT: Methods for additional analyses, such as subgroup analyses and adjusted analyses

In addition, we performed a sub-analysis of the IOP changes over the study period in those patients who did not report any changes in the amount of anti-glaucoma ophthalmic solution used during the study. The IOP profiles were compared in the NIA and IA groups. The IOP changes from the pre-IA to the post-IA period were also compared in the IA groups.

RESULTS

13a) CONSORT: For each group, the numbers of participants who were randomly assigned, received intended treatment, and were analysed for the primary outcome

One-hundred ninety-four patients were randomly assigned to the two groups, and 81 IA and 90 NIA patients completed the study.

13b) CONSORT: For each group, losses and exclusions after randomisation, together with reasons

These data are depicted in Figure 1 and Table 1.

13b-i) Attrition diagram

One-hundred ninety-four patients were randomly assigned to the two groups, and 81 IA and 90 NIA patients completed the study.

14a) CONSORT: Dates defining the periods of recruitment and follow-up

Registration for the GSS began in 2005. By 2008, only ophthalmologists were allowed to browse the registered medical records and the patients were required to view their registered medical records in the presence of the attending ophthalmologist at the clinic.

Internet access by the patients began in 2008. We randomly selected the patients for Internet access between January 2008 and December 2008.

14a-i) Indicate if critical "secular events" fell into the study period

14b) CONSORT: Why the trial ended or was stopped (early)

NA

15) CONSORT: A table showing baseline demographic and clinical characteristics for each group

In Table 1.

15-i) Report demographics associated with digital divide issues

In Table 1

16a) CONSORT: For each group, number of participants (denominator) included in each analysis and whether the analysis was by original assigned groups

16-i) Report multiple "denominators" and provide definitions

In Figure 1

16-ii) Primary analysis should be intent-to-treat

The number of anti-glaucoma ophthalmic solution used during the study period significantly increased in the NIA group (($p<0.001$ for the right eye, $p= 0.03$ for the left eye) but not in the IA group. The percentages of patients with unchanged, increased, and decreased anti-glaucoma ophthalmic solution use during the study period were, 61.1%, 17.8%, and 3.3%, respectively, in the NIA group, and 56.8%, 8.6%, and 13.6%, respectively, in the IA group ($p < 0.001$).

17a) CONSORT: For each primary and secondary outcome, results for each group, and the estimated effect size and its precision (such as 95% confidence interval)

NA

17a-i) Presentation of process outcomes such as metrics of use and intensity of use

17b) CONSORT: For binary outcomes, presentation of both absolute and relative effect sizes is recommended

NA

18) CONSORT: Results of any other analyses performed, including subgroup analyses and adjusted analyses, distinguishing pre-specified from exploratory

The Internet access significantly altered the IOP trend from increasing to decreasing in the IA group ($P=0.002$) among the patients who did not have any medication changes.

18-i) Subgroup analysis of comparing only users

19) CONSORT: All important harms or unintended effects in each group
The patients in this study were GSS users. Given that many patients cannot use a personal computer or the Internet due to their age, poverty, or other reasons, it is possible that the patients in this study are not representative of general glaucoma patients. The present study did not examine how frequently the patients accessed the GSS.
19-i) Include privacy breaches, technical problems
The GSS is based on the concept of an information security management system.
All of the data transferred from medical charts to the GSS are managed offline using locally developed data management programs.
The users were asked to replace their temporary passwords and to register their user names with the system. To maintain patient information security, no information that could identify a patient, such as name, age, sex, telephone number, home address, or business address, was accessible through the Internet.
19-ii) Include qualitative feedback from participants or observations from staff/researchers

DISCUSSION

20) CONSORT: Trial limitations, addressing sources of potential bias, imprecision, multiplicity of analyses
20-i) Typical limitations in ehealth trials
The patients in this study were GSS users. Given that many patients cannot use a personal computer or the Internet due to their age, poverty, or other reasons, it is possible that the patients in this study are not representative of general glaucoma patients. The present study did not examine how frequently the patients accessed the GSS. Therefore, further investigations should be performed to verify the relationship between the frequency of use and the improvements in glaucoma treatment associated with using the system.
21) CONSORT: Generalisability (external validity, applicability) of the trial findings
21-i) Generalizability to other populations
The patients in this study were GSS users. Given that many patients cannot use a personal computer or the Internet due to their age, poverty, or other reasons, it is possible that the patients in this study are not representative of general glaucoma patients.
21-ii) Discuss if there were elements in the RCT that would be different in a routine application setting
The patients in this study were GSS users. Given that many patients cannot use a personal computer or the Internet due to their age, poverty, or other reasons, it is possible that the patients in this study are not representative of general glaucoma patients.
22) CONSORT: Interpretation consistent with results, balancing benefits and harms, and considering other relevant evidence
22-i) Restate study questions and summarize the answers suggested by the data, starting with primary outcomes and process outcomes (use)
The main finding of this study was that self-browsing clinical data through the GSS may improve the effectiveness of medication among glaucoma patients.
22-ii) Highlight unanswered new questions, suggest future research
The patients in this study were GSS users. Given that many patients cannot use a personal computer or the Internet due to their age, poverty, or other reasons, it is possible that the patients in this study are not representative of general glaucoma patients. The present study did not examine how frequently the patients accessed the GSS. Therefore, further investigations should be performed to verify the relationship between the frequency of use and the improvements in glaucoma treatment associated with using the system.

Other information

23) CONSORT: Registration number and name of trial registry
UMIN000006982
Study for searching usage status and effectiveness of supporting system for chronic diseases
24) CONSORT: Where the full trial protocol can be accessed, if available
NA
25) CONSORT: Sources of funding and other support (such as supply of drugs), role of funders
None

X26-i) Comment on ethics committee approval
This study was performed in accordance with the Helsinki Treaty and was approved by the University of Yamanashi Ethical Review Board.
x26-ii) Outline informed consent procedures
Written informed consent was obtained from all of the patients.
X26-iii) Safety and security procedures
The GSS is based on the concept of an information security management system.
The users were asked to replace their temporary passwords and to register their user names with the system. To maintain patient information security, no information that could identify a patient, such as name, age, sex, telephone number, home address, or business address, was accessible through the Internet.
X27-i) State the relation of the study team towards the system being evaluated
None
