# Supplementary figures and images for: Impact of Patient Access to Internet Health Records on Glaucoma Medication: Randomized Controlled Trial
Source: J Med Internet Res. 2014 Jan 15;16(1):e15. doi: 10.2196/jmir.2795 (PMC3906702; doi:10.2196/jmir.2795)

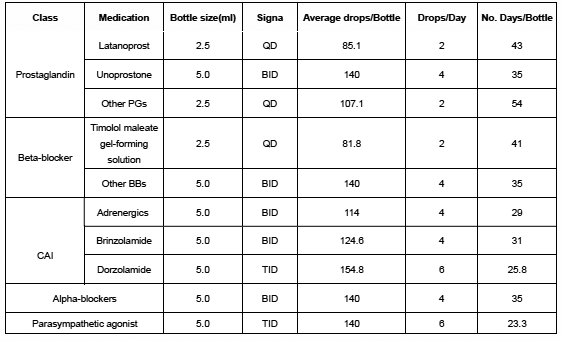

Supplement: Supplementary file 2 [file jmir_v16i1e15_app2.tif]

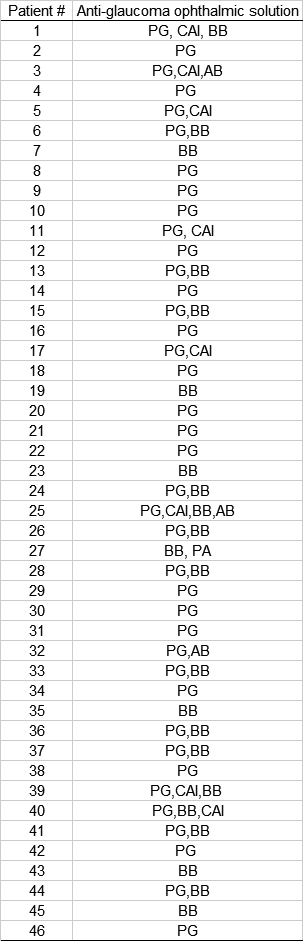

Supplement: Supplementary file 3 [file jmir_v16i1e15_app3.tif]

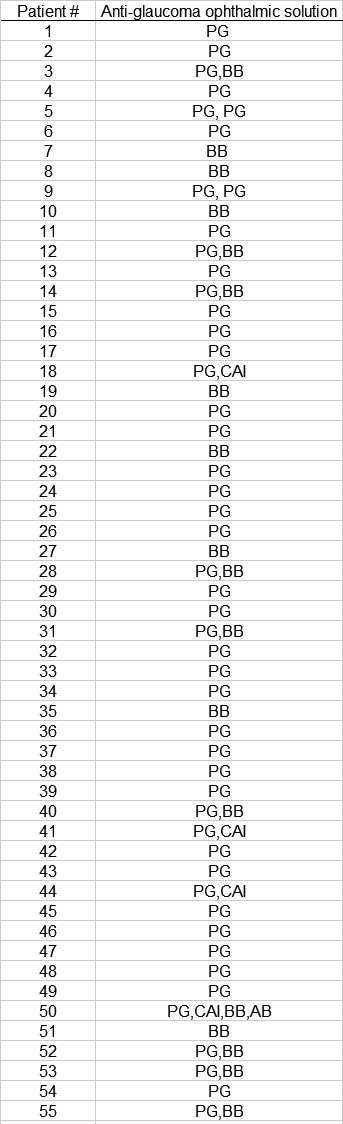

Supplement: Supplementary file 4 [file jmir_v16i1e15_app4.tif]
